# Electronic Health Record (EHR)-Based Community Health Measures: An Exploratory Assessment of Perceived Usefulness by Local Health Departments

**DOI:** 10.1186/s12889-018-5550-2

**Published:** 2018-05-22

**Authors:** Karen F. Comer, P. Joseph Gibson, Jian Zou, Marc Rosenman, Brian E. Dixon

**Affiliations:** 1 Doctoral student, Department of Health Policy and Management, Indiana University Richard M. Fairbanks School of Public Health, Indianapolis, USA; 2 Director of Collaborative Research and Health Geoinformatics, The Polis Center at Indiana University-Purdue University, Indianapolis, USA; 3 Director of Epidemiology, Marion County Public Health Department, Indianapolis, USA; 4 Assistant Professor, Department of Mathematical Sciences, Worcester Polytechnic Institute, Worcester, MA USA; 5 Associate Professor of Pediatrics, Northwestern University Feinburg School of Medicine, Chicago, USA; 6 Research Scientist, Regenstrief Institute, Indianapolis, USA; 7 Associate Professor, Department of Epidemiology, Indiana University Richard M. Fairbanks School of Public Health, Indianapolis, USA; 8 The Polis Center at Indiana University-Purdue University Indianapolis (IUPUI), 1200 Waterway Blvd, Indianapolis, Indiana, 46202 USA

**Keywords:** Community health assessment, Community health measure, Electronic health record, Health information exchange, Population health, Public health

## Abstract

**Background:**

Given the widespread adoption of electronic health record (EHR) systems in health care organizations, public health agencies are interested in accessing EHR data to improve health assessment and surveillance. Yet there exist few examples in the U.S. of governmental health agencies using EHR data routinely to examine disease prevalence and other measures of community health. The objective of this study was to explore local health department (LHD) professionals’ perceptions of the usefulness of EHR-based community health measures, and to examine these perceptions in the context of LHDs’ current access and use of sub-county data, data aggregated at geographic levels smaller than county.

**Methods:**

To explore perceived usefulness, we conducted an online survey of LHD professionals in Indiana. One hundred and thirty-three (133) individuals from thirty-one (31) LHDs participated. The survey asked about usefulness of specific community health measures as well as current access to and uses of sub-county population health data. Descriptive statistics were calculated to examine respondents’ perceptions, access, and use. A one-way ANOVA (with pairwise comparisons) test was used to compare average scores by LHD size.

**Results:**

Respondents overall indicated moderate agreement on which community health measures might be useful. Perceived usefulness of specific EHR-based community health measures varied by size of respondent’s LHD [F(3, 88) = 3.56, *p* = 0.017]. Over 70% of survey respondents reported using community health data, but of those < 30% indicated they had access to sub-county level data.

**Conclusion:**

Respondents generally preferred familiar community health measures versus novel, EHR-based measures that are not in widespread use within health departments. Access to sub-county data is limited but strongly desired. Future research and development is needed as LHD staff gain access to EHR data and apply these data to support the core function of health assessment.

**Electronic supplementary material:**

The online version of this article (10.1186/s12889-018-5550-2) contains supplementary material, which is available to authorized users.

## Background

Public health professionals lack timely information to track population health status. They also lack sub-county data, data aggregated to geographic levels smaller than county, to plan geographically-targeted interventions to improve population health. [1] Population health measures are usually based on birth and death certificates or surveys. [2] Birth and death certificates capture data on nearly everyone, but only at limited, discrete points in individuals’ lives. Surveys can fill in the gap, but only for a fraction of the population, often limiting their ability to provide reliable information about specific sub-populations or geographic areas. Furthermore, surveys collect data infrequently. [3]

To monitor and improve community health, public health professionals need more inclusive, frequently updated information about population health. For community health assessment and improvement planning, the use of the smallest geographic unit possible improves the identification of relevant local assets and resource gaps. [4] Public health agencies often lack the resources to collect the volume and frequency of data necessary to monitor and address health at a sub-county level using surveys alone.

The increased adoption of electronic health record (EHR) systems by health care providers provides an opportunity for access to more timely data, [5, 6] while the integration of geographic information system (GIS) technology with EHR data provides the opportunity to create small area views of community health. [7, 8] Few health departments in the U.S. have routine access to EHR data or integrated GIS with EHR data. However, two recent studies, one by a state health agency [3] and one by a local health department [9], compared EHR-based community health measures of chronic disease prevalence with those from the Behavioral Risk Factor Surveillance System (BRFSS), a widely used population health survey in the U.S. Both studies concluded that EHR-based prevalence measures were similar to BRFSS, thereby increasing interest among health departments with respect to using EHR data to augment existing health assessment activities.

While EHR data have potential, it remains unclear which measures will be the most useful or reliable for routine population health assessment. In our work to examine reliability of EHR-based community health measures, we sought to understand the perceived usefulness of community health measures by those who would ultimately consume them – public health professionals. Exploring the information needs [10, 11] of end users is an important aspect of informatics, an information science that has many applications within the field of public health. [12] Moreover, public health lacks consensus on the set of measures that agencies should use for community health assessment, [1] and the most commonly used measures do not currently make use of EHR data. [13] Therefore, before examining reliability of EHR-based community health measures, we sought to identify a broad set of measures to target for our research.

In this article, we present the results of an exploratory survey of public health workers that sought to illuminate the perceived usefulness of various community health measures. The survey examined measures that might be useful at a sub-county level, as these measures can be challenging to derive from nationally representative datasets collected across states and regions. The article summarizes the rationale behind the study, the development of the study instruments and measures, and it discusses the findings from respondents on the frontlines of local public health.

## Methods

To explore the perceived usefulness of community health measures, we conducted an online survey of local public health professionals. Survey items addressed the usefulness of specific community health measures, while also collecting background information on respondents’ current access to sub-county population health data (e.g., data aggregated at the ZIP code, census tract, or neighborhood level), current and desired uses of sub-county data, and which sub-county geographic units were most relevant to respondents’ work.

### Theoretical framework

The underlying theoretical framework guiding this research is the Technology Acceptance Model (TAM), an information science framework that seeks to explain the determinants of an information system’s use following its introduction into an organization. [14] The TAM includes a number of determinants, including perceived usefulness, which is defined as the degree to which a person (e.g., public health worker) believes that using a particular information system or a given health measure would enhance his or her job performance. [15] The TAM and perceived usefulness have been extensively studied in the fields of management science, operational research, and informatics. This includes numerous studies examining the introduction of health information systems in clinical and public health organizations. [16, 17] Our goal was to apply perceived usefulness as a construct, independent of the broader TAM, as we did not seek to understand usage following the introduction of a new system but the potential value of community health measures to individuals working in local health departments. Measuring perceived usefulness of a potential measure would enable us to design an information system that could produce measures perceived to be useful, thereby facilitating adoption of the eventual information system when introduced into a public health agency.

### Participants

Using a convenience sample, the survey targeted local public health agency workers throughout Indiana. The Indiana State Department of Health emailed the questionnaire to an administrator at each local health departments (LHD) in Indiana (*n* = 93). In addition, the questionnaire was distributed via local public health listservs, including those of the Indiana Public Health Training Center (n = ~ 3000) and the Community Health Engagement Program of the Indiana Clinical and Translational Sciences Institute (n = ~ 700). Finally, the questionnaire was sent directly to the epidemiology staff at the Marion County Public Health Department (MCPHD) (*n* = 10). Survey recipients were invited to forward the survey link to other individuals in their agencies, including administrators, directors, and managers of community programs and services, health communications and education specialists, public health nurses and nurse case managers, environmental health specialists, vital records managers, epidemiologists, data analysts, statisticians, GIS analysts, and social workers.

The survey was fielded for 6 weeks from late April to early June 2014 following distribution of the email invites, and invitees received one reminder e-mail in week 3.

### Survey development

To populate the survey instrument, we compiled an initial list of fourteen (14) community health measures based on HEDIS (Healthcare Effectiveness Data and Information Set). HEDIS measures were selected because they generally can be generated quickly at a community level. HEDIS measures were designed to be drawn from medical records [18] for measuring hospital performance, and they include several wellness and disease management measures relevant to infectious and chronic diseases. [8] For example, there are HEDIS measures for the proportion of women who are screened for chlamydia, the proportion of eligible individuals who received colorectal cancer screening, and blood pressure control measures for patients with hypertension. We hypothesized that LHD professionals would be interested in using these measures to better understand the burden of and prevention efforts relevant to diseases such as chlamydia, asthma, and diabetes. [19–21]

To identify additional measures of interest for measuring the health of geographic populations, a member of the study team polled epidemiologists and department heads at MCPHD, the largest health department in Indiana. This resulted in the identification of an additional ten (10) measures, for a total of twenty-four (24) potential measures. This was subsequently reduced to a total of twenty-three (23) potential measures because one of the HEDIS-based measures (colorectal cancer screening) was already understood to be unavailable because it is handled generally as an outpatient procedure and does not necessarily get captured by the EHR.

We also drafted a set of fifteen questions. Two Likert-scale questions were included: one to measure perceived usefulness of each potential HEDIS measure and the other to identify population characteristics of most interest (e.g., age, sex, race, socioeconomic status). In addition, questions were included to collect information about respondent demographics, current use of sub-county community health measures, and geographic level(s) of interest.

The initial questionnaire was pilot tested by staff at MCPHD and at the Polis Center at IUPUI. Pilot testers were asked to complete the draft survey and identify questions or terms that were unclear. Their feedback was used to modify the questionnaire prior to distribution to the sample population, but their responses were excluded from the final dataset. The final questionnaire is included as an additional PDF file [see Additional file 1].

### Analysis of survey data

Survey responses were imported into SPSS Statistics (IBM Corporation, Armonk, NY) for analysis. Analysis excluded responses from persons who did not report that they worked at an LHD.

Scoring of Likert scale responses on the usefulness of proposed measures was applied as follows: 3 = very useful, 2 = somewhat useful, 1 = not very useful, 0 = not at all useful. Average usefulness scores were calculated for each measure and stratified by size of the respondent’s local health department, since the more specialized staff in larger LHDs may generate uses of community health measures that would be low priority in small LHDs. The size of an LHD was classified as small (< 10 employees), medium (11–50 employees), large (51–250 employees), or very large (251–1000 employees). This classification is routinely used by the National Association of City and County Health Officials (NACCHO) to analyze LHD data. Average scores were coded based on quartile, ranging from “Most useful” (3) to “Least useful” (0).

A one-way ANOVA (with pairwise comparisons) test was conducted to compare average scores by LHD size. The significance of the differences in the average scores by LHD size was determined using both Tukey and Fisher’s multiple comparison methods.

Using feedback from LHD respondents, in conjunction with an internal assessment of which measures have EHR data readily available, we constructed a final set of 11 community health measures that were both desired and feasible.

## Results

A total of 133 responses were received from LHD staff in Indiana. With survey responses from 31 different county health departments, one-third (33%) of Indiana’s 92 LHDs had some degree of representation in the survey results. Survey responses were geographically diverse. The largest number of respondents worked in Central Indiana, the region with the highest concentration of citizens and public health professionals. Given an estimated 2400 FTE public health employees working in Indiana LHDs, the response rate was approximately 4–6%, depending on whether the respondents were full-time or part-time. [22]

Respondents were somewhat evenly divided among LHDs that were small (< 10 employees), medium (11–50 employees), large (51–250 employees), and very large (251–1000 employees), with 25% (*n* = 33), 29% (*n* = 39), 25% (n = 33), and 21% (*n* = 28), respectively.

Environmental health and health communication/education (26% each) and senior administration/executive (23%) were the most commonly reported LHD roles. Slightly more than 10% reported a role in vital records (11%) or public health nursing (10%), while an even smaller percent reported a role in data analysis (8%).

### Differences in usefulness scores

Average usefulness scores for each potential measure are represented in Table 1. The average scores, based on *all* LHD responses to this particular question (*N* = 91), ranged from 1.12 to 2.17 for the different measures. When stratified by health department size, the resulting average scores for each potential measure ranged from 0.86 to 2.50.

**Table 1:** Perceived Usefulness of Potential Community Health Measures

	Average Scores of Perceived Usefulness^a^ by LHD Size^b^				
Potential Community Health Measure	All (*n* = 91, 30 LHDs)	Small (n = 22, 14 LHDs)	Medium (*n* = 23, 11 LHDs)	Large (*n* = 24, 4 LHDs)	Very Large (*n* = 22, 1 LHD)
1. Vaccination Coverage School-age Children	2.17	2.50	2.30	1.81	2.05
2. Flu Vaccination Coverage	2.07	2.45	2.17	1.55	2.10
3. Prevalence Substance Abuse	2.06	2.27	2.00	1.82	2.14
4. Hepatitis B and/or Hepatitis C	2.05	2.32	2.26	1.82	1.76
5. Diabetes Prevalence	2.03	2.23	2.13	1.81	1.95
6. HPV Vaccination Coverage	1.99	2.18	2.17	1.48	2.09
7. Chlamydia/Gonorrhea/Syphilis Incidence	1.92	2.10	1.96	1.64	2.00
8. Hypertension and Other Common Cardiovascular Disease Prevalence	1.92	2.24	1.91	1.62	1.91
9. Asthma and COPD Prevalence	1.91	2.18	1.78	1.50	2.18
10. Depression Prevalence	1.85	1.90	1.55	1.87	2.09
11. HIV Screening	1.83	1.95	1.86	1.52	1.95
12. Various Cancers Incidence	1.82	2.14	1.64	1.68	1.82
13. Evidence of Violence or Trauma	1.75	1.82	1.68	1.57	1.91
14. Chlamydia Screening	1.69	1.76	1.78	1.48	1.73
15. Cholesterol Screening Patients with Cardiovascular Conditions	1.69	2.00	1.83	*1.43*	1.48
16. Breast Cancer Screening	1.67	2.05	1.55	1.45	1.64
17. Hemoglobin A1c (HbA1c) Testing, Diabetic Patients	1.53	1.64	1.70	*1.33*	*1.43*
18. ER Use by Asthmatics	1.49	1.55	1.24	1.18	2.00
19. Cholesterol Levels (LDL-C) < 100 mg/dL Cardiovascular Patients	1.46	1.90	*1.43*	*1.24*	*1.25*
20. Hemoglobin A1c (HbA1c) Controlled < 8 Percent, Diabetic Patients	*1.29*	1.57	*1.23*	*1.14*	*1.24*
21. Dental Caries Prevalence	*1.22*	*1.41*	*.91*	*1.20*	*1.38*
22. Asthma ADHD Prevalence Comorbidity Impact on ED Visits	*1.15*	*1.36*	*.91*	*.90*	*1.41*
23. ER Use by People with Dental Pain or Infections	*1.12*	*1.23*	*.86*	*1.00*	*1.36*

^a^Usefulness (Avg Scores) Quartile 1 (Most useful): 2.00–2.50 Quartile 2: 1.77–1.99 Quartile 3: 1.45–1.76 *Quartile 4 (Least useful): 0.86–1.44*

^b^LHD Size Small: < 10 employees Medium: 11–50 employees Large: 51–250 employees Very Large: 251–1000 employees

**Fig. 1:**
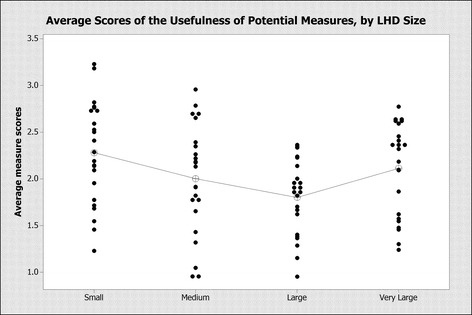
Average scores of the usefulness of potential measures, by LHD size

Respondent answers varied significantly based on LHD size [F(3, 88) = 3.56, *p* = 0.017]. Average measure scores were normally distributed, without notable heteroscedasticity, consistent with requirements for a valid ANOVA analysis. Small, medium, and very large LHDs generally ranked measures as more useful than large LHDs. While there is not a big difference between the average scores of small and medium LHDs or between small and very large LHDs, the average scores of small and very large LHDs are significantly higher than the average scores for large LHDs (Fig. 1: Average Scores of Usefulness of Potential Measures).

LHD Size (# of employees): Small (< 10), Medium (11–50), Large (51–250), Very Large (251–1000).

### Integrating perceived usefulness with ready access of measure data

Our synthesis of respondent perceptions and internal assessment of available EHR data in shown in Table 2. Several measures perceived as “most useful,” such as immunization rates, had to be excluded from our selection for pilot implementation because the data necessary for measure calculation are not available in EHR systems at the community level. Furthermore, some measures perceived as “somewhat useful” or “least useful” were included, because they are readily available in EHR data or routinely generated for HEDIS reporting. All HEDIS measures were selected for pilot implementation, because they can be generated quickly at a community level.

**Table 2:** Synthesis of Respondent Perceptions and Internal Assessment of Available Data in EHRs

Potential Community Health Measure	Perceived Usefulness^a^	Readily Available in EHRs	Selected for Pilot Implementation
1. Vaccination Coverage School-age Children^c^	Most Useful	No	No
2. Flu Vaccination Coverage^c^		No	No
3. Prevalence Substance Abuse^c^		No	No
4. Hepatitis B and/or Hepatitis C^c^		Yes	No
5. Diabetes Prevalence		Yes	Yes
6. HPV Vaccination Coverage^c^	Useful	No	No
7. Chlamydia/Gonorrhea/Syphilis Incidence		Yes	Yes
8. Hypertension and Other Common Cardiovascular Disease Prevalence		Yes	Yes
9. Asthma and COPD Prevalence		Yes	Yes
10. Depression Prevalence		Yes	Yes
11. HIV Screening^c^		Yes	No
12. Various Cancers Incidence		Yes	No
13. Evidence of Violence or Trauma^c^	Somewhat Useful	No	No
14. Chlamydia Screening		Yes	Yes^d^
15. Cholesterol Screening Patients with Cardiovascular Conditions		Yes	Yes^d^
16. Breast Cancer Screening		Yes	Yes^d^
17. Hemoglobin A1c (HbA1c) Testing, Diabetic Patients		Yes	Yes^d^
18. ER Use by Asthmatics		Yes	No
19. LDL-C Levels < 100 mg/dL for Patients with Cardiovascular Conditions		Yes	Yes^d^
20. Hemoglobin A1c (HbA1c) Controlled < 8 Percent, Diabetic Patients	Least Useful	Yes	Yes^d^
21. Dental Caries Prevalence^c^		No	No
22. Asthma ADHD Prevalence Comorbidity Impact on ED Visits^c^		No	No
23. ER Use by People with Dental Pain or Infections^c^		Yes	No
24. Colorectal Cancer Screening	Not measured^b^	No	No

^a^Category derived from Quartiles in Table 2

^b^Not included in the survey

^c^These measures were proposed by epidemiologists and department heads at MCPHD

^d^These measures are commonly reported by health systems as part of the Healthcare Effectiveness Data and Information Set (HEDIS) to payers

### Access to and use of Sub-County community health data

While the majority (71%) of the 133 respondents reported using community health data, only 27% (*n* = 36) reported having access to those data at the sub-county level. Thirty-five percent (*n* = 47) reported not having access, 16% (*n* = 21) reported being unsure of whether they had access, and 22% (29) did not respond. Of those with access, 92% (*n* = 33) of respondents reported on their current use of sub-county data. Roughly similar proportions used it for community health needs assessment, identifying high-risk groups, health improvement planning, targeting interventions, and identifying disparities. Of those who did not report current access (*n* = 97), 71% (*n* = 69) reported on their desired use (Table 3: Use of Sub-County Data). At least 64% (*n* = 44) and up to 73% (*n* = 50) indicated they would use such data, if available, to identify high-risk groups (73%), target interventions (67%), assess community health needs (64%), support health improvement planning (64%), or improve routine public health functions (64%). A smaller percentage indicated they would use it to identify disparities (52%) or for program evaluation (49%).

**Table 3:** Use of Sub-County Data

Sub-County Data Use	Use by those with access (*n* = 33)	Desired use by those without access (*n* = 69)
	% (n)	
For community health needs assessment	61% (20)	64% (44)
For health improvement planning	55% (18)	64% (44
To identify high-risk groups	55% (18)	73% (50)
To target interventions to appropriate populations	52% (17)	67% (46)
To identify disparities	46% (15)	52% (36)
For program evaluation	36% (12)	49% (34)
For improvement of routine public health functions	36% (12)	64% (44)
I do not use the available sub-county data.	6% (2)	7% (5)
Other	9% (3)	

### Interest in population characteristics

Age and socio-economic status were the population characteristics of highest interest to LHD respondents (Table 4: Interest in Population Characteristics). Race/ethnicity, education, and gender were also of great interest, with over 50% of the 90 respondents who answered this question indicating that these were of either “Highest priority” or “High priority”. Other characteristics respondents noted as being of interest included health insurance coverage, marital status, and refugee and immigrant status.

**Table 4:** Interest in Population Characteristics

Population Characteristic	Highest priority	High priority	Of interest	Not applicable	No response
*n* = 90	% (n)	% (n)	% (n)	% (n)	% (n)
Age	45.6 (41)	34.4 (31)	13.3 (12)	2.2 (2)	4.4 (4)
Socioeconomic Status	36.7 (33)	31.1 (28)	17.8 (16)	6.7 (6)	7.8 (7)
Race/Ethnicity	23.3 (21)	25.6 (23)	21.1 (19)	15.6 (14)	14.4 (13)
Gender	17.8 (16)	21.1 (19)	30 (27)	16.7 (15)	14.4 (13)
Education	14.4 (13)	32.2 (29)	27.8 (25)	10.0 (9)	15.6 (14)
Disabled	7.8 (7)	20.0 (18)	37.8 (34)	13.3 (12)	21.1 (19)
Sexual Orientation	6.7 (6)	8.9 (8)	32.2 (29)	30.0 (27)	22.2 (20)
Veterans	1.1 (1)	17.8 (16)	34.4 (31)	23.3 (21)	23.3 (21)
Other	1.1 (1)	4.4 (4)	0 (0)	15.6 (14)	78.9 (71)

## Discussion

Using a convenience sample of LHD professionals in a single state via an online survey, we sought feedback on a list of potential population health measures hypothesized to be useful to community health assessment. The feedback was leveraged to prioritize measure selection for a research project that aimed at generating reliable population health measures using EHR data. A modest response rate yielded feedback balanced across LHDs of various sizes. The feedback provided useful guidance for directing the research project. The results further offer several important findings relevant to the perceived usefulness, access and use of population health measures by LHD workers.

The survey indicated a lack of access to but strong interest in sub-county data for common LHD activities such as community health needs assessment, identifying high-risk groups, and community health improvement planning. While two-thirds of the respondents indicated that they used community-level data, just one-third reported access to information at the sub-county level. To best use their limited human and financial resources, LHDs need information about sub-areas within their jurisdiction. This is especially important to identify and monitor health inequity, which prior research has demonstrated to vary widely based on geography. [23–26] These results confirm both the strong interest in but current lack of access to data that would enable LHDs to better target efforts aimed at improving health for specific geographic regions or sub-populations. Future studies should generate and evaluate the use of sub-county measures by LHD workers in the context of surveillance, policy, and community health assessment.

A second finding is the lack of consensus among LHDs with respect to the perceived usefulness of the proposed population health measures. While respondents indicated some agreement on which measures might be more useful than others (e.g., average ratings trended in the same direction), there were notable differences in the average ratings between small versus large LHDs and between very large and large LHDs. Smaller LHDs, in general, rated most measures as more useful than larger LHDs. This pattern might be explained, in part, by the fact that larger LHDs tend to possess more data collection resources (e.g., locally developed community surveys, larger staff sizes) and may therefore be able to generate their own health indicators. They may perceive EHR-based indicators as less useful than those generated by themselves. Still the fact that LHD size may influence the perceived usefulness of an indicator is noteworthy, especially for those seeking to design or produce health indicators of interest to LHDs. Given these findings, qualitative methodologies such as focus groups or interviews may be a way to understand the reasons behind different perceptions. Furthermore, future studies may test different sets of measures to meet the varying needs of LHDs based on size and function.

A third noteworthy finding is measures rated most useful were those most familiar to LHD respondents, or measures available from sources other than EHRs. Vaccination rates are calculated by state-based immunization information systems; notifiable disease rates are calculated by LHDs as well as state health agencies from mandatory case reporting; non-communicable disease rates are assessed using population health surveys; and cancer incidence is reported by state tumor registries from mandatory hospital reporting. Health care utilization and quality measures, such as emergency room utilization among patients with dental pain and the proportion of patients with diabetes under control, were seen as less desirable.

A focus on the familiar is notable for two reasons. First, this result surprised the research team which expected LHD respondents to perceive indicators to which they currently do not have access as more useful than indicators they routinely capture and use. One reason LHDs may perceive the familiar as more useful is that public health professionals have learned how to make good use of what is available, such as prevalence of notifiable diseases. Familiar indicators from a new source might also be perceived more favorably because they generate deeper understanding of a disease than a new indicator for which LHDs may not have a reference. Finally, these indicators are recommended for community health assessment by the U.S. Centers for Disease Control and Prevention, [13] which might have influenced respondents’ selections.

In addition, health process and quality indicators, which might be unfamiliar to public health professionals, are those that are most readily available from EHRs. EHR systems routinely generate indicators such as the number of patients with diabetes who had a glycosylated hemoglobin measured within the last 12 months. These indicators are reported to insurance companies including the U.S. Centers for Medicare and Medicaid Services, and several are aligned with the Robert Wood Johnson Foundation’s Culture of Health measures. [27] Yet these indicators are absent from initiatives like the County Health Rankings, which tend to favor health survey data. [28] Sharing these indicators with public health departments would require minimal work for health systems, which is in contrast to the desire of LHDs to have EHRs begin sending more detailed information on notifiable diseases such as symptoms associated with Hepatitis C. [29] It is this type of mismatch that can lead to frustration in conversations with EHR vendors as well as health system leaders, and this type of mismatch is the right type for public health leaders to consider when planning for information systems used within the LHD. [30] Information system designers should pay attention to such mismatches as they contemplate not where systems are today but where they are headed given the changing culture of health in the U.S.

Finally, these findings are important for the design and implementation of information systems that facilitate bi-directional exchange of data between clinical and public health organizations. Soliciting input from public health professionals via the survey is one form of what is broadly described as user-centered design (UCD). UCD processes seek to involve users in decision-making with respect to how a work process or information system is designed to function. [31] In this case, we sought input on which indicators would be most useful as the research team sought to develop something that would inform practice. Although other UCD methods such as focus groups are sufficient, we chose a survey in order to cast a broad net as we hope to disseminate our tools and products across a wide range of LHDs.

The findings illustrate the importance of UCD, because otherwise our choice of indicators and product design would have been driven primarily by the study team and its very large LHD partner. Based on the differences in opinion among LHDs, we would not have been able to capture these differences via a focus group at one or two LHDs. The insights provided by survey responses were instrumental in helping the team balance the desires of the research team (e.g., focus on indicators largely not used by LHD workers today) with the perceived needs of real LHD staff (e.g., focus on indicators that are familiar to LHD workers).

### Limitations

This study has several limitations to note. First, self-selection of respondents may have introduced bias. Although the proportion of health departments of different size responding was similar to the actual proportion, those LHDs who self-selected to respond may have different uses and preferences than their counterpart health departments. The methods used to solicit responses are more akin to a convenience sampling technique rather than a stratified random sample. Furthermore, the sample size was small compared to the number of local public health professionals in Indiana, and the responding LHDs may not be representative of Indiana as a whole. It is also likely that some individuals received the questionnaire more than once. It was not possible to assess the amount of duplication as the organizations that distributed our survey have a policy against sharing their membership lists. No measures were used to prevent an individual from taking the survey twice because the research team judged it highly unlikely by that a public health professional would do so.

Second, analysis of the data was limited to descriptive statistics; therefore, causal relationships should not be inferred. Future studies of information needs should consider larger samples of LHDs as well as the use of power analysis to enable more robust quantitative exploration across the size spectrum of LHDs.

### Implications for Policy & Practice

There are few examples in the U.S. where governmental health agencies use EHR data to examine population health. It is important to gather evidence on how EHR systems in health care organizations can improve the practice of epidemiology and community health. This study adds knowledge regarding those EHR-based community health measures perceived to be most useful to public health workers in local health departments. The results can be used as input to future initiatives that aim to generate needed community health measures using EHR data and make them available to frontline public health workers.

## Conclusion

A survey of LHD respondents yielded valuable feedback for a project seeking to leverage EHR data to generate and share community health measures with LHDs charged with assessing community health. Perceptions vary among LHDs based on their size, suggesting that a one-size fits all approach may not be sufficient. Furthermore, LHD respondents tend to prefer indicators that are familiar as opposed to novel indicators not in widespread use among public health professionals. The results also suggest that current access to sub-county data is limited but strongly desired. These observations suggest pathways for future research and application development as LHD staff become more familiar with accessing and using EHR data to support the core function of health assessment.

## Additional file

Additional file 1 Final questionnaire (the survey instrument) (PDF 287 kb)
