# Supplementary material for: Electronic Health Record (EHR)-Based Community Health Measures: An Exploratory Assessment of Perceived Usefulness by Local Health Departments
Source: BMC Public Health. 2018 May 22;18:647. doi: 10.1186/s12889-018-5550-2 (PMC5964696; doi:10.1186/s12889-018-5550-2)
Supplement: Supplementary file 1 — Final questionnaire (the survey instrument) (PDF 287 kb) [file 12889_2018_5550_MOESM1_ESM.pdf]

Measuring Community Health Using Electronic Health Records

This survey includes 15 or fewer questions and should take you approximately 5-10 minutes to complete. Please read the informed consent information below and then indicate whether you would like to proceed.

IRB STUDY # 1402792577

INDIANA UNIVERSITY INFORMED CONSENT FORM FOR
Prioritization of community health measures via survey of community health sector professionals

Principal Investigator (PI): Brian E. Dixon, MPA, PhD, School of Informatics, Indiana University, 317.278.0046, bedixon@regenstrief.org

You are invited to participate in a research study to help us to help identify and prioritize community health measures based on data from electronic health records (EHRs) from multiple providers around the State of Indiana. You were selected as a possible subject as a member of the health sector. We ask that you read this form and ask any questions you may have before agreeing to be in the study.

The study is conducted by researchers at Indiana University and funded by Robert Wood Johnson Foundation.

STUDY PURPOSE
The purpose of this study is to identify community health measures that could inform whether newer sources of data will enable improved assessment of population health.

NUMBER OF PEOPLE TAKING PART IN THE STUDY:
If you agree to participate, you will be one of approximately 500 subjects who will be participating in this research.

PROCEDURES FOR THE STUDY
If you agree to be in the study, you will be asked to complete a survey, either online or paper copy survey. The survey will require approximately 5-10 minutes of your time. The survey will ask questions that gather information about the size of your organization, the extent to which community health data is used in your job, type of data worked with on a daily basis, and your prioritization of community health measures.

RISKS OF TAKING PART IN THE STUDY:
While on the study, the risks are:
Minimal risk is associated with participating in this research. Some people feel that providing information on a survey is an invasion of privacy and are uncomfortable answering some questions.

BENEFITS OF TAKING PART IN THE STUDY:
The benefits to participation that are reasonable to expect are:
By providing your input about the community health measures you have the potential to contribute to our understanding of the social and environmental determinants of health.

ALTERNATIVES TO TAKING PART IN THE STUDY:
Instead of being in the study, you have these options:
The only alternative is not participating.

CONFIDENTIALITY
Efforts will be made to keep your personal information confidential. Electronic survey responses and any printed versions of survey results will be securely stored and accessible only to the principal investigator and staff directly involved in this project. Survey results will be destroyed following completion of the project (expected date: 12/31/2015). Anonymity will be maintained as names will not be collected in the course of this research and there will be no direct identification of subjects. Any identifying information, such as names of organizations, etc, will be removed from the survey results prior to analysis.

We cannot guarantee absolute confidentiality. Your personal information may be disclosed if required by law. Your identity will be held in confidence in reports in which the study may be published. Organizations that may inspect and/or copy your research records for quality assurance and data analysis include groups such as the study investigator and his/her research associates, the Indiana University Institutional Review Board or its designees, the study sponsor, the Indiana CTSI, and (as allowed by law) state or federal agencies, specifically the Office for Human Research Protections (OHRP), the National Institutes of Health (NIH), etc., who may need to access research records.

PAYMENT
You will not receive payment for taking part in this study.

CONTACTS FOR QUESTIONS OR PROBLEMS
For questions about the study, please contact Brian Dixon (317.278.0046). For questions about your rights as a research participant or to discuss problems, complaints or concerns about a research study, or to obtain in

1. Would you like to proceed with this survey?

- ◯ Yes
- ◯ No

Measuring Community Health Using Electronic Health Records

SECTION 1 – Demographics

2. For which type of organization do you work? Choose the one that best applies. If none apply, please specify your organization type after "Other".

- ◯ Local health department
- ◯ State health department
- ◯ Hospital
- ◯ Clinic or community health center
- ◯ Community-based organization

Other (please specify)

[ ]

3. Approximately how many employees are there in your organization?

- ◯ < 10
- ◯ Between 11 and 50
- ◯ Between 51 and 250
- ◯ Between 250 and 1000
- ◯ > 1000
- ◯ I don't know

4. What is your role in the organization? Check all that apply.

- ☐ Senior administration
- ☐ Marketing
- ☐ Community benefit
- ☐ Health communication/education
- ☐ Environmental health
- ☐ Vital records
- ☐ Social work
- ☐ Epidemiological analysis/data analysis/statistics
- ☐ Geographic information analysis

Other (please specify)

[ ]

Measuring Community Health Using Electronic Health Records

SECTION 2 - Community Health Data

* 5. For your job, do you work with or use community health data?

- ◯ Yes
- ◯ No
- ◯ I don’t know

6. Do you currently have access to measures of community health for sub-county areas (e.g., disease rates by neighborhood, health access by ZIP code, screening rates by census tract)?

- ◯ Yes
- ◯ No
- ◯ I don't know

Measuring Community Health Using Electronic Health Records

7. Please describe some sub-county measures you have seen or worked with for your job. (Sub-county is a geographic area smaller than a county, e.g. zip code, census tract, etc.)

8. For what tasks do you use these sub-county data? Check all that apply.

- ☐ To identify disparities
- ☐ To identify high-risk groups
- ☐ To target interventions to appropriate populations
- ☐ For program evaluation
- ☐ For community health needs assessment
- ☐ For health improvement planning
- ☐ For improvement of routine public health functions
- ☐ I do not use the available sub-county data.

Other (please specify)

Measuring Community Health Using Electronic Health Records

9. If sub-county community health data were available to you, for what tasks would you likely use these data? Check all that apply.

- [ ] To identify disparities
- [ ] To identify high-risk groups
- [ ] To target interventions to appropriate populations
- [ ] For program evaluation
- [ ] For community health needs assessment
- [ ] For health improvement planning
- [ ] For improvement of routine public health functions
- [ ] I would not use sub-county data if it were available

Other (please specify)

Measuring Community Health Using Electronic Health Records

10. Which sub-county area(s) are most meaningful to your agency's work? Check all that apply.

- [ ] Neighborhood
- [ ] Census block group (Average population of ~1,500)
- [ ] Census tract (Average population of ~4,000)
- [ ] ZIP code (Average population of ~8,000)
- [ ] I don't know

Other (please specify)

Measuring Community Health Using Electronic Health Records

11. How do you typically define neighborhood for purposes of data analysis? Check all that apply.

- [ ] By the street boundaries specified by local government or neighborhood groups.
- [ ] As a collection of geographic units (e.g. block groups, census tracts, or ZIP codes) that most closely matches specified street boundaries
- [ ] Based on distance from a neighborhood landmark.
- [ ] Based on cumulative population surrounding a neighborhood landmark.
- [ ] Based on clusters of geographic units with similar socio-demographic characteristics
- [ ] I don't know

Other (please specify)

Measuring Community Health Using Electronic Health Records

12. Please indicate which population characteristics are of highest interest to you or your organization. Choose up to three for each level of priority.

	Highest priority	High priority	Of interest	Not applicable
Age	○	○	○	○
Gender	○	○	○	○
Race/Ethnicity	○	○	○	○
Sexual orientation	○	○	○	○
Education	○	○	○	○
Socio-economic status	○	○	○	○
Disabled	○	○	○	○
Veterans	○	○	○	○
Other	○	○	○	○

Other (please specify)

13. For each the following community health measures, please indicate how useful it is to your job.

	Very useful	Somewhat useful	Not very useful	Not at all useful
Prevalence of diabetes	○	○	○	○

	Very useful	Somewhat useful	Not very useful	Not at all useful
Hemoglobin A1c testing for patients with diabetes	◯	◯	◯	◯
Hemoglobin A1c controlled at <8% for patients with diabetes	◯	◯	◯	◯
Prevalence of asthma and chronic obstructive pulmonary disease	◯	◯	◯	◯
Emergency room utilization for people with asthma	◯	◯	◯	◯
Prevalence of asthma among those with attention deficit hyperactivity disorder / impact of co-morbidity on emergency department visits	◯	◯	◯	◯
Prevalence of hypertension and other common cardiovascular diseases	◯	◯	◯	◯
Cholesterol screening for patients with cardiovascular conditions	◯	◯	◯	◯
Cholesterol levels < 100 mg/dL for patients with cardiovascular conditions	◯	◯	◯	◯
Breast cancer screening	◯	◯	◯	◯
Incidence of various cancers	◯	◯	◯	◯
Prevalence of depression	◯	◯	◯	◯
Incidence of chlamydia, gonorrhea, and syphilis	◯	◯	◯	◯
Chlamydia screening	◯	◯	◯	◯
HIV screening	◯	◯	◯	◯
Prevalence of hepatitis B and/or hepatitis C	◯	◯	◯	◯

	Very useful	Somewhat useful	Not very useful	Not at all useful
Vaccination coverage for school age children (2-dose (MMR) Measles, Mumps, Rubella; DTaP; 2 dose varicella)	○	○	○	○
Flu vaccination coverage	○	○	○	○
HPV vaccination coverage	○	○	○	○
Emergency room utilization by people with dental pain/infections	○	○	○	○
Prevalence of dental caries	○	○	○	○
Evidence of violence/trauma (e.g., domestic violence)	○	○	○	○
Prevalence of substance abuse	○	○	○	○

14. What additional community measures, if any, do you think would be important for policymakers to consider in the future?

15. Comments:
